# Care and services partnership in Quebec birthing centres: myth or reality?

**DOI:** 10.1186/s12884-024-06362-w

**Published:** 2024-03-07

**Authors:** Justine Sales, Louise Normandin, Marie-Pascale Pomey

**Affiliations:** 1 Université Claude Bernard Lyon 1, Lyon, France; 2 Research Centre, University of Montreal Hospital Centre, Montréal, QC Canada; 3 Centre d’excellence sur le partenariat avec les patients et le public, Montréal, QC Canada; 4 Department of Health Policy, Management and Evaluation, School of Public Health, University of Montréal, 7101 Av du Parc 3e étage, Montréal, QC H3N 1X9 Canada; 5 Department of Family Medicine and Emergency, University of Montreal, Montréal, QC Canada

**Keywords:** Women & family partnership, Birthing centres, Care relationship, Midwife, Continuum of care, Women-centered care

## Abstract

**Context:**

Working with women to best meet their needs has always been central to midwifery in Quebec, Canada. The creation of birthing centres at the end of the 1990s consolidated this desire to prioritize women’s involvement in perinatal care and was intended to encourage the establishment of a care and services partnership between care providers and users. The aim of this pilot study is to evaluate the perceptions of clients, midwives and birth assistants of the way in which women are involved in partnership working in Quebec birthing centres.

**Methods:**

A single qualitative case and pilot study was conducted with midwives (*n* = 5), birth assistants (*n* = 4), a manager (*n* = 1), clients (*n* = 5) and members of the users’ committee (*n* = 2) at a birthing centre in Quebec, Canada in July and August 2023. The partnership was evaluated using the dimensions of a validated CADICEE questionnaire.

**Results:**

The women and professionals stressed that the relationship was established in a climate of trust. The caregivers also attached importance to autonomy, information sharing and decision-making, adaptation to context, empathy and recognition of the couple’s expertise. The women confirmed that they establish a relationship of trust with the professionals when the latter show empathy and that they adapt the follow-up to their knowledge and life context. Key factors in establishing this kind of care relationship are the time given, a de-medicalized environment, the comprehensive care received, and professionals who are well-informed about the partnership. In addition, the birthing centre has a users’ committee that can put forward ideas but has no decision-making powers.

**Conclusions:**

Both the women and the professionals at the birthing centre appear to be working in partnership. However, at the organizational level, the women are not involved in decision-making. A study of all birthing centres in Quebec would provide a more comprehensive picture of the situation.

**Supplementary Information:**

The online version contains supplementary material available at 10.1186/s12884-024-06362-w.

## Context

The beginning of the 21st century was marked by a worldwide movement to involve patients in their healthcare. The province of Quebec, Canada led the way in this transformation of healthcare systems by proposing a model based on partnership [1]. The “Montreal model” proposes a continuum of patient involvement, from information to partnership in various areas [1]. This model of care is based on recognizing patients’ experiential knowledge as complementary to the scientific knowledge of their professionals [2]. The profound changes brought about by such partnerships are part of a wider movement toward the democratization of health care and the empowerment of patients and citizens in health. Patient engagement can help improve population health and the quality of care and services [3–10]. In perinatal care, there is a paucity of literature on how care partnership can be introduced or what its effects are [11], even though the care model has evolved considerably in recent years, particularly in Quebec.

In the province of Quebec, women can have their pregnancy monitored either in a hospital or a clinic by doctors or advanced practice nurses, or in a birthing centre (BC) by midwives (MWs). In 2021, the proportion of midwife-attended births amounted to only 4.3% of all births [12]. There are 20 BCs in the province, including 6 in Montreal [13]. Quebec’s health insurance reimburses up to 100% of expenses for pregnancy follow-ups in BCs. To ensure their safety, women with pre-existing chronic illnesses or who develop obstetric or fetal pathologies during pregnancy or childbirth are referred to a specialized hospital, which may be a university-affiliated hospital or an Integrated Health and Social Services Network (CISSS) or an Integrated Health and Social Services University Network (CIUSSS) with which the BC is associated [14]. With a few exceptions, BCs are the only places where midwives practise in Quebec. They provide pregnancy, childbirth and postpartum care. In BCs, clients are monitored by one or two midwives throughout the perinatal period. Birth assistants (BAs) support midwives during childbirth and assist women in the immediate postpartum period. When monitored by a BC, births can take place in the BC, at home or in the associated hospital.

The history of BCs in Quebec is closely linked to that of midwives and women. In the 1970s, the women’s movement in Quebec challenged the medicalization of pregnancy and childbirth [15]. Women called for the legalization of the midwife profession so that they could be monitored and give birth outside of hospitals [16]. As early as 1990, BC pilot projects were set up to study the safety of the practice of midwifery (*Act respecting the practice of midwifery within the framework of pilot projects*, R.S.Q., c. P-16.1) [17]. In 1998, midwifery was recognized as a safe profession, and the law of September 1999 legalized the profession of midwife (*Midwives Act*, S.Q. 1999, c. 24, s. 82) [14, 17]. This practice was legalized to meet a need expressed by women and their partners [15]. The Quebec BCs’ provincial committee created a reference framework to define what a BC is [14]: it is a “living environment” where midwives adapt to the needs of families in order to offer humanized and comprehensive pregnancy care [14]. In this way, the participation of women and their spouses in the care and organization of services became recognized as an essential part of meeting families’ needs [14]. But, paradoxically, women and their partners were referred to as clients, similar to the practice of the Canadian Association of Midwives. In 2015, BCs in Montreal confirmed the importance of the involvement of women and their partners as one of their fundamental values: “The participation of parents, to whom the establishment confers real resources adapted to their reality, is the very foundation of the community character of a BC.“ [18]. Unfortunately, COVID-19 significantly impacted Canadian midwives due to lack of recognition, drastic increases in their workloads, and even moral distress that exacerbated burnout [19]. In particular, the pandemic protocols limited their ability to provide emotional support and develop the deeper relationships with clients, which they say is “the essence of midwifery care” [19]. As a result, BCs came to be seen as a privileged place for a care and services partnership with women during the perinatal period.

The aim of this study is to evaluate the perceptions of clients, midwives and birth assistants of the way in which women are involved in the partnership approach in the BC. The specific objectives are to describe: (1) obstacles and levers to the implementation of the care relationship and women’s involvement in their care, (2) the various ways in which women are involved in the organization and governance of the BC, and (3) the obstacles to women’s involvement in the organization and governance of BCs.

## The birthing centre

The BC used for this study was one of the first BCs in Quebec, created at a time when there were only pilot projects. It is located in Montreal. In terms of governance, the BC Board’s executive committee consists of five people: the Chief Executive Officer (CEO) of the CIUSSS, the Head of Midwifery Services (Manager), the Chair (a MW), a Vice-Chair (a MW) and a Secretary (a MW). This committee is the BC’s decision-making body and liaises with the Administrative Committee of the CIUSSS. Eleven midwives and six BAs work there. As far as clients are concerned, the BC does not apply any territorial restrictions. 7.6% of its clients are in vulnerable situations (without health insurance, asylum seekers, or in very precarious social and financial situations) [20]. From March 2022 to March 2023, 244 women were monitored and there were 149 deliveries (10% in hospital, 12% at home and 78% in the BC) [20]. One of the special features of this BC is that it offers courses, given by the BAs, on parenthood throughout the pre and postnatal period. Lastly, the BC has a users’ committee composed of five members. Because of the COVID-19 pandemic and its restrictions, the committee was disbanded before being reconstituted in 2020.

## Materials and methods

A single in-depth qualitative case study was carried out in July and August 2023 based on 17 semi-structured interviews with clients and professionals at the BC in Montreal, Canada [21, 22]. The aim of this pilot study was to describe the care relationship and the various ways in which women are involved in their follow-up.

### Populations

Two populations were studied. The first was the professionals at the BC (*n* = 10), including the MWs, the BAs and the manager. The second group (*n* = 7) consisted of clients^1^ who either were in pregnancy or postpartum follow-up (*n* = 5) or were former clients and members of the BC users’ committee (*n* = 2).

All the participants were invited to take part in an interview by videoconference or in person in an office in the BC. The 11 MWs and 6 BAs working at the BC were invited by the manager. The invitations were accepted by 5 MWs out of 11 and 4 BAs out of 6. Clients were recruited in person at the BC by either the manager or a member of the research team. The inclusion criteria were: to be French speaking and either a current pregnancy or postpartum woman client of the birthing center or a member of its users’ committee. The exclusion criteria were: to be under 18 years of age, to have refused to participate in the study, and to be unable to give consent. A total of 15 clients were approached and 7 accepted.

### Data collection

All respondents agreed to participate by signing an informed consent form before the interview, and all the interviews were recorded. The interviews were conducted by people with experience in qualitative research (JS, LN, MPP).

The interviews focused on the seven dimensions of the validated CADICEE tool, which measures the degree of partnership of care and services between patients and caregivers [23]: (1) Confidence, (2) Autonomy, (3) (participation in) Decision-making, (4) (sharing) Information, (5) personal Context, (6) Empathy, and (7) Expertise. The professionals were also asked about obstacles and levers to implementing the care relationship (Appendix 1). The clients were also asked questions on why they chose to be followed up at the BC, the strengths of the follow-up, areas for improvement and their involvement with the BC. If the client was also a member of the parents’ committee, her role and responsibilities were discussed (Appendix 2).

In addition, a meeting was held with the BC manager to discuss: (1) the decision-making procedures at the BC, (2) the resources available to professionals for establishing a partnership relationship with clients, (3) the role of the users’ committee, and (4) the obstacles and levers to implementing the care and service partnership relationship (see Appendix 3).

At the end of the interviews, questions were asked to build brief demographic descriptions of the professionals (age, length of practice) and the clients (age, level of education, number of children).

### Analysis

Data analysis involved the following steps [24]: (a) in-depth readings of the first two verbatim transcripts and identification of key ideas, (b) development of a preliminary analytic framework based on both the CADICEE framework, using a deductive approach [23], and the nature of the information discussed in the interviews [25], and (c) a constant comparison of codes. This led to the development of the final coding scheme. Two researchers (JS and MPP) co-developed the codebook, conducted the thematic analysis independently in QDA Miner Lite v2.0 software (Provalis Research) [26], and then discussed which verbatim transcripts best illustrated the themes to be developed in the manuscript.

## Results

### Characteristics of respondents

We conducted 17 semi-structured interviews with clients and professionals at the BC.

Ten semi-structured interviews were conducted with the midwives (*n* = 5), the BAs (*n* = 4) and the manager (*n* = 1). These professionals were all women, with an average age of 36 and an average length of practice of 4.5 years. One of the midwives is Vice-Chair of the executive committee of the BC’s Board, one of the BAs is the organization’s support person for the users’ committee, and one of the BAs is also a student in midwifery.

Seven semi-structured interviews were conducted with clients (*n* = 5) and members of the users’ committee (*n* = 2) (Table 1). Their average age was 36 years. They were all university-educated. Of the women whose pregnancies were being monitored, half were in their second trimester and the other half were in their third.

**Table 1:** Clients’ characteristics

	Pregnancy follow-up (*n* = 4)	Postpartum follow-up (*n* = 1)	Members of the BC users’ committee (*n* = 2)
Accompanied by their partner (*n* = 2)	2	0	0
Primiparous (*n* = 3)	1	1	1
Multiparous (*n* = 4)	3	0	1
Second follow-up in the BC (*n* = 2)	2	0	0
Not a Canadian citizen (*n* = 2)	2	0	0
Experienced transfer to a hospital (*n* = 2)	1	0	1

### Interviews

The interviews lasted an average of 39 min (ranging from 21 to 57 min – median of 40 min).

Verbatim transcripts from the interviews were classified into three themes: (1) the quality of the care relationship, (2) the obstacles and levers to the implementation of the care relationship for the women in their follow-up, (3) the organization and governance of the BC.

The verbatim transcripts presented are anonymized but attributed according to point of view: a pregnancy client (PC) or a postpartum client (PPC), a midwife (MW), a birth assistant (BA), or a manager (MG).

### The quality of the care relationship

Seven main themes emerged from the interviews related to the quality of the care relationship: (a) confidence, (b) autonomy, (c) decision-making, (d) information sharing, (e) personal context, (f) empathy, and (g) expertise.

#### Confidence

For most of the clients, the care relationship with their professionals was mainly based on confidence: “*It’s a great relationship. I really trust them, and that’s certainly the most important thing.*” (PC5). Trust was often seen as an essential aspect of this relationship, in particular giving a boost to clients’ self-confidence: “*They responded, they took the time to respond, and that gave me confidence in the system, in her, but also in myself*.” (PC4). For carers, it also appears to be the basis of the care relationship: *“It’s a relationship based essentially on trust. It’s on the basis of a relationship of trust that we’re able to build everything else*.” (MW3).

This trust can be established in particular through the importance given to and respect for consent and the choices made by clients.

Just the fact that the midwives say, ‘Is it okay if I touch your tummy?’ So just asking at the beginning made a big difference. (PC4)

It’s always about offering, and respecting the answer, too. (BA1)

#### Autonomy

As part of the philosophy of their profession, the midwives attach great importance to the autonomy of families.

For me, it’s very important. And for me, that’s the primary objective of midwifery care: empowering women and families before natural or physiological childbirth in fact. (MW5)

#### Decision-making

In particular, the freedom to make decisions gives families a sense of autonomy.

All my choices were discussed, so we were able to decide at every stage whether any care needed to be given or any additional tests needed to be carried out, and this was always with our agreement. So yes, we did feel quite autonomous. (PC3)

But while some families feel completely autonomous, others can be unsettled: *“I arrived expecting to be taken by the hand, but that’s not what happened. And it threw me off balance.”* (PC4).

#### Sharing information

The sharing of information emerged as an essential part of the monitoring process in the BC. The professionals seek to provide clear information to enable families to make informed choices, without ever influencing or interfering in the decision-making process:

Every aspect was presented to us. I really had the impression that it was decision support and not pressure. (PC4)

Each examination, test or treatment modality is presented with its advantages and disadvantages, and then discussed so that families can make their own decisions.

I always have a choice in everything. She explains, for example: last time, we were talking about the diabetes test. I thought it was an obligation, that it came out of the blue, but then she said, ‘No, no, it’s your choice, we’ll talk about it.’ (PC9).

#### Personal context

The BC follow-up was widely described by the women as more “humanized”, often justifying their choice to be monitored there. Medical parameters do not seem to be the only considerations when monitoring a pregnancy:

Wow, there’s a much more human side to - it’s not just a medical thing, where you feel like a number. (PC4)

The care relationship in the BC is based on adapting care to the personal context of each family’s life. The midwives offer women personalized care by adjusting it to their wishes or previous deliveries.

If we talk about this birth, if we have clearly talked about the previous birth and thought about how we could take into account what was good or not so good in order to adapt to the new situation, yes. (PC3)

In other words, I don’t have the same people in front of me all the time. I always have different people, so it’s a matter of listening. (MW3)

At the BC, pregnant women are considered in the context of their wider lives, as their spouses and children are invited to attend throughout the pregnancy. Spouses appear to be fully involved in pregnancy monitoring, childbirth and the postpartum period.

The team also suggested that my first child should come to the visits. (PC5)

It was the quality of the support, the time they devoted, the space that was given to the spouse, which wasn’t the same at all with a doctor. (PC4)

The few spouses who were present with their wives during the interviews were able to point out the attention they had received at the BC:

I feel totally integrated into the conversations. (PC9)

In order to be able to adapt to each of their clients’ life contexts, the carers are very open-minded, meaning that the monitoring is not only centered on medical concerns, but can also take into account the clients’ religion and the people around them:

Our first meeting with the midwife involved a question. She asked us, “Do you have any kind of faith? Are there things you should know about childbirth? (PC2)

#### Empathy

Professionals place considerable emphasis on empathy, which the families are generally aware of and satisfied with:

Empathy towards us, I’ve always had the impression that all the staff, both the students and when we called, there was this empathy towards us. (PC4)

Like multiparous women, some women often have expertise in the perinatal field. Taking into account their experiential knowledge and skills is part of the follow-up in the BC.

I still had enough knowledge and, on the other hand, my husband has no siblings, no experience. But we both felt that we were being listened to at our level, and that we were bringing our expertise to the table. (PC2)

It’s really important to recognize the knowledge they already have. And we can see that when it’s a third child or a first child, we know that they will call on us less often because they’re able to recognize the baby’s signs. (BA1)

#### Expertise

Recognizing women’s expertise fosters a care relationship based on equality. Several carers emphasized this desire to achieve an equal relationship with the couples, in which each party passes on their knowledge:

There really is a notion where the aim is to put our baggage of knowledge together, and that’s where the notion of an egalitarian link comes in. (MW5)

### Obstacles and levers to the implementation of the care relationship

Several levers were identified for fostering a relationship based on partnership in the BC: (a) time, (b) environment, (c) a small team, (d) a trained team and (e) the context of pregnancy.

#### Time

The one cited most often by professionals and women was time. A consultation in the BC lasts 45 to 60 min. MWs are able to present all the information they need to make independent decisions, learn more about their clients’ wishes and needs, and take the time to answer their questions.

There was a lot more time for me at the birthing centre. A lot more opportunity for me to ask questions. They took the time to make sure I had the opportunity to ask all my questions. (PPC1)

The fact that we have a lot of time means that we can explain things. (MW2)

If you have concerns and you call, then they talk on the phone, you know, for 1 h, even if you don’t have an appointment. They really have the time to answer your questions. (PC2)

#### Environment

A second lever identified by the majority is the environment of the BC, often described as warm and welcoming. Housed in a former presbytery, the BC seeks to preserve as much as possible the spirit of a family home, with minimal medical care.

And I also liked the birthing centre environment. I found it warmer. (PC1)

#### A small team

Throughout their care, women will meet with a limited number of carers. Only two or three midwives will accompany them during their pregnancy, their childbirth and the postpartum period. The professionals get to know the women better: their personal history, and their wishes, needs and concerns.

And there was also more continuity: I saw the same midwife. I was with the same midwife until 30–36 weeks, and then switched off with the other midwife. So I really liked that I knew it was one or the other for the birth, not just any family doctor or obstetrician who was on call that evening. (PC1)

You know, we’re a small team. The birthing centre coordinator, she’s here with us. I think size plays a big part. (BA2)

#### A trained team

In addition, most of the midwives and BA interviewed were able to define a patient partnership relationship and had the necessary training.

It’s definitely at the heart of the philosophy of midwifery practice in Quebec, so it’s passed on to us from the beginning of our studies, then afterwards, and it’s something we learn to put into practice on a daily basis in the birthing centre. (MW5)

#### Pregnancy context

Lastly, pregnancy is not a pathology, and in the majority of cases is part of a life project in which preparations are made to welcome a child. This particular context could encourage a relationship based on partnership:

Childbirth is a special moment in this person’s life, it’s a unique moment. Maybe it’s the only time she’s ever given birth, or maybe the second. It’s very special. (MW4)

Some obstacles to a partnership-based care relationship were also identified: (a) communication, (b) a change in the BC’s clientele, and (c) transfers.

#### Communication

First, in certain situations access to services in the client’s mother tongue is difficult to obtain. Although the BC does its utmost to match couples and midwives according to the language they speak, some couples were unable to take advantage of this opportunity, and communication problems arose. These situations were more common among the spouses, as it limited their integration into the follow-up program.

Apart from the fact that I want to say that my midwife who doesn’t speak Spanish is a bit difficult, obviously especially for my partner. And it’s funny that my partner pointed out to me, ‘Ah, I don’t know what the point is of me being here. [laughs]. (PC11)

#### A change in the clientele

Historically, the women followed-up in the BC are committed and informed women who fought for these rights alongside the midwives. But since 2020, the BC has also been receiving clients who are in vulnerable situations (without health insurance, asylum seekers, or in positions of great social and financial precariousness). This change in clientele could represent an obstacle to partnership. These clients often have needs that are quite different from what the carers have encountered to date.

Given the language and cultural barriers, it’s a slightly less balanced partnership, I’d say. In the sense that they’re less likely to seek autonomy. You know, they mostly want a healthy baby. (MW3)

#### Transfers

Finally, transfers between the BC and the hospital appear to be an obstacle to partnership. During transfers, midwives accompany their clients to the hospital for the postpartum follow-up, for example following a major tear during childbirth. While midwives are accustomed to preparing women for this situation, the transfer is still a challenge when it comes to building a partnership-based care relationship, especially when the transfer takes place just before delivery.

I think that if it’s during delivery, the midwife transfers with the client, but if it’s just a few hours before, my transfer was really from one system to another with no follow-up, and so it’s still disorientating. (CG5)

### BC organization and governance

Governance of the BC is organized around the Midwife Board, whose executive committee is made up of midwives, the MW manager and the CEO of the CIUSSS. The Midwife Board has set up a number of committees to assess quality of practice and to train and maintain skills in order to improve client care and services, including the perinatal committee and the midwifery clinical procedures drafting committee. No client or spouse sits on any of the committees:

We don’t have a patient partner on all our committees, so it might be interesting to systematize this. (MG)

Clients are also involved in the users’ committee: the parents’ committee. Reconstituted in the wake of the pandemic, this committee is made up of five women who gave birth at the BC. “*We are five women who have just given birth at the birthing centre over the last few years*.” (CPP1). The BC provides a BA, herself a former client, who initiated the committee and helps with organizational matters: “*We found it very helpful to have a birth assistant in charge of organization, so she can facilitate and act as a link between these more active members*.” (MG) There is no formal hierarchical organization. There are no spouses on the committee. Its current functioning dates back to the end of the pandemic. These users organize picnics and talks for parents and parents-to-be on topics such as “preparing siblings for the arrival of the baby” or pelvic physiotherapy after childbirth: “*Otherwise the families organized a picnic, which was super popular. It’s brand new. It was really up to them.*” (MG).

The parents’ committee has no influence on decision-making at the BC: “*Not so much. […] It’s not so much a reflex that they participate in all decisions. But maybe it could be relevan*t.” (MG) Members can submit ideas for the continuous improvement of services offered at the BC:

It’s really interesting, because the issue of the day is all about gender. I find it very valuable too, because it means we’re constantly readapting to the needs of families. (MG)

In addition, the committee members interviewed were in favor of establishing co-management, and felt that the BC would be willing to listen to them and include them in governance. The BC manager appeared to be in favor of starting to include them:

I could be interested. Then I feel that at home I really have an openness. You know if I did something like that, I’m sure I’d be listened to, then I’d learn a lot. (PPC2)

However, one of the obstacles to the involvement of women and their spouses is the lack of information about the parents’ committee:

I wouldn’t know, actually. I’d say I don’t have any information about it.” (PC5) and the lack of time during pregnancy or postpartum: “I feel I’m running out of time in my life management. (PC11)

## Discussion

This study is the first, to our knowledge, to be carried out in the province of Quebec to assess perceptions of the partnership-based relationship among BC clients and healthcare professionals. The study sought the perceptions not only of professionals, but also of women in pregnancy and postpartum care, primiparous and multiparous women, and women who are members of the users’ committee.

The results show that clients and professionals have fairly similar perceptions of the quality of the care relationship, based on the partnership principles of confidence, autonomy, decision-making and sharing of information, adaptation to personal context, empathy and recognition of expertise [23]. The time given to clients and their partners, a de-medicalized environment, comprehensive follow-up and professionals who are informed about the partnership have all been identified as levers for implementing such a care relationship. However, the women’s participation at the organizational level remains fairly modest; it is more at the level of information exchange and consultation.

At the clinical level, the vision developed in the reference framework for BCs [14] is well understood and integrated into practices by the health professionals. It is perceived as implemented by the women who are being followed there. In fact, the MWs and BAs generally agree to establish a relationship of trust with their clients and demonstrate empathy. They give the women and their families the time they need to consider all the facets of their lives, and this is greatly appreciated by the women. This climate fosters the exchange of information on both sides and, for clients, the ability to make informed decisions. These results are consistent with the literature, in which collaboration with women and their partners to best meet their needs is considered to be at the heart of midwifery practice in BCs [27].

However, when examined more closely, a few points of divergence emerge between these two visions. The clients’ perceptions of continuity of care differ from the perceptions of their professionals. Indeed, they are more sensitive to a change of MW during pregnancy, which can lead to different relationships with the two MWs. They are also more affected by transfers to hospital. From the professionals’ point of view, these transfers are carried out optimally, but the clients reported a lack of continuity between the two structures, and would like the MW to be more present during the transfer, as well as during their hospital care. Transitional moments are often difficult events, and require special attention [28]. In addition, this could be explained by the difficulties encountered integrating MWs into hospitals [29]. The MWs have complete access to the hospital with which their BC is associated. However, they usually face a lack of interest among the healthcare professionals and differences with them in terms of philosophy and scope of practice [29]. Another discrepancy concerns the possibility of using the language of one’s choice during consultations. Several clients mentioned that their spouses felt excluded from the conversation because they did not speak French well. While MWs could be selected on the basis of their ability to express themselves in certain languages, this approach does not seem to be systematically implemented.

With regard to fostering partnership at the clinical level, our study highlights factors that are less frequently found in the literature as being the most important [1]. For example, one of the factors most often identified in the literature concerns the importance of having sufficient human resources, whereas in this case the number of resources appears to be appropriate, allowing the time needed to build a partnership relationship. The role played by the environment is noted as important, but this is rarely mentioned in the literature [5, 30]. In this setting, the non-medicalized environment was seen as one of the key factors in fostering partnership. These results illustrate the importance of considering the role of architecture as a key factor in fostering partnership [1].

In terms of organization, the BC has set up a parents’ committee to gather suggestions on how to improve services. However, women are still not included on improvement committees or in governance to directly influence decision-making [11]. These results diverge from those found in the literature, which reports partnerships at the organizational rather than at the clinical level in other fields, and particularly in Quebec [4]. This may be linked to the midwifery philosophy of care, which is based on the humanization of care and a holistic vision of the services offered, on the one hand, and with the ancestral presence of peer support to accompany childbirth, on the other [15, 16]. However, BCs’ governance might change because of Bill 15, recently proposed by Quebec’s Minister of Health, Christian Dubé. For example, Bill 15 would have medical directors head BCs [31]. The professional association of midwives in Quebec (The Regroupement Les sages-femmes du Québec) feels that this bill will impact MWs’ autonomy, creating medical subservience, and encourage a pathological approach to pregnancy and childbirth rather than global and humanized care [32].

### Limitations

The main limitation of this pilot study is that it was carried out in a single BC, which makes it impossible to generalize the results [21, 33]. A multiple-case study of several Quebec BCs would be needed to confirm our results. A mixed-methods study could be considered, using a context-specific version of the CADICEE questionnaire to measure women’s level of involvement in their care in the BC [23]. The number of spouses interviewed was insufficient for an in-depth study of the care relationship with them, whereas data saturation was achieved in the client interviews. Professionals and clients of the studied BC did not report any actual and major impacts of COVID-19 on the quality of care or the human resources. However, the consequences of the pandemic might still influence the organization of BCs and the partnership between clients and midwives in care and governance. Lastly, the women interviewed had a fairly high level of education, and in the vast majority were Caucasian. A future study should seek out the views of a more diverse cohort of women.

### Reflexivity

There was no particular relationship between the main researchers and the BC or the midwifery staff. Regarding the choice of BCs in Montréal, they were all contacted. The centre selected was the first one to respond and agree to participate.

Even though one of the researchers is a midwife, she had never practised in this country and did not have any assumptions about how midwifery is practised in this special context. In addition, the other researchers did not have a background in midwifery and did not have any prior assumptions or beliefs about the study. They focused on their working hypothesis based on the literature and tried to confirm or reject it.

Even so, the authors, who come from three different specialties, had regular reflections and discussions during the data collection, and analysis and when interpreting the data in order to avoid personal biases.

### Recommendations

In light of our findings, and in agreement with the MW manager, we are proposing a number of recommendations to strengthen the partnership at both the organizational and clinical levels.

At the organizational level, we recommend:

- Incorporating two parents on the BC Board’s executive committee, so that they can be involved in decision-making;
- Strengthening the role played by the parents’ committee, so that it is called upon more regularly to address the various issues facing the BC;
- Publicizing the parents’ committee to pregnant women and their partners, so that they will be aware of its existence; and.
- Creating a continuous service improvement committee, composed at least of two parents, which could support the executive committee and make suggestions to the parents’ committee on areas for improvement.

At the clinical level, we recommend:

- Organizing a meeting at the beginning of the process with the two midwives who will be monitoring the parturient, so that the woman knows them from the outset and is followed by the one who most closely meets her needs;
- Encouraging follow-up by an MW who speaks the mother tongue of the woman and/or her partner, to promote communication with both of them; and.
- Reviewing transfer arrangements with clients, to ensure that they are accompanied by an MW during hospital transfers.

## Conclusion

Partnership in care to best meet women’s needs has always been at the heart of midwifery practice in Quebec, and this heralded the worldwide movement to involve patients in their health care. In view of the information reported by both the professionals and their clients, there appears to be care partnerships at the BC we studied. As far as partnership at the organizational level is concerned, there are some areas for improvement that could easily be addressed. A study of all birthing centres in Quebec would provide a more comprehensive picture of the situation.

### Electronic supplementary material

Below is the link to the electronic supplementary material.

Supplementary Material 1

## Data Availability

All authors had full access to the data and materials. The data is available from the authors upon reasonable request.

## Abbreviations

BC: Birthing centre
MW: Midwife
CIUSSS: Centre Intégré Universitaire de Santé et de Services Sociaux or Integrated Health and Social Services University Network
CISSS: Centre Intégré de Santé et de Services Sociaux or Integrated Health and Social Services Network
BA: Birth assistant
CEO: Chief Executive Officer
PC: Pregnancy client
PPC: Postpartum client
MG: Manager

## Footnotes

1 The authors recognize that not all the pregnant people identifiy as women.
