# Supplementary material for: Care and services partnership in Quebec birthing centres: myth or reality?
Source: BMC Pregnancy Childbirth. 2024 Mar 7;24:177. doi: 10.1186/s12884-024-06362-w (PMC10918890; doi:10.1186/s12884-024-06362-w)
Supplement: Supplementary file 1 — Supplementary Material 1 [file 12884_2024_6362_MOESM1_ESM.docx]

**Appendices**

**Appendix 1. Interview Guide for Professionals**

| Question | Questions for further development |
| --- | --- |
| What is the relationship between clients and MWs/BAs at the birthing centre? | What do you consider to be the most important aspect of this relationship? |
| Depending on the answer to the previous question, follow up with these questions: | |
| How important do you consider the role played by trust in this relationship? | - Mutual trust?  - How do you ensure that the women and their families trust you?  - Can you identify any factors specific to the birthing centre environment that help or hinder the establishment of a relationship of trust between you and the women? |
| How important do you consider the role played by the autonomy of the women and their families? | - How do you manage to empower women and families?  - Can you identify any factors specific to the birthing centre environment that promote or limit the autonomy of women and their families? |
| Do you involve the women and their partners in the decision-making about their health and aftercare? | - How do you involve the women and their partners in the decision-making about their health and aftercare?  - How important is shared decision-making to you?  - What factors specific to the birthing centre environment can you identify that encourage or limit decision-making by the women and their families? |
| How important do you consider the role played by sharing information with women and their families? | - How do you share information with the women and their families about their health and care?  - What factors specific to the birthing centre environment can you identify as facilitating or limiting the sharing of information with women and their families? |
| Do you adapt your (midwifery) practice to the context of women's lives and the desires and needs of the women and their families? | - How do you adapt your (midwifery) practice to the context of women's lives and the desires and needs of the women and their families?  - How do you draw up the birth plan?  - How is it important to you?  -What factors specific to the birthing centre environment can you identify as helping or hindering this adaptation? |
| How important do you consider the role played by empathy in your practice? | - What factors specific to the birthing centre environment can you identify that encourage or limit your empathy? |
| Do you feel that your clients have useful expertise, knowledge and experience that, with your support, they can draw on for their pregnancy, childbirth and postpartum? | - What role do you play in your practice in recognizing and mobilizing your clients' knowledge and expertise?  - How do you mobilize this expertise? |
| Do you have all the resources you need to establish a care and service partnership with your clients and their families? | - Are you satisfied with the level of commitment you can offer to the families?  - What resources are involved? |
| Are you familiar with the notion of the concept of a care and service partnership with clients and caregivers? | Can you define it? |
| Is there anything you would like to share that we did not discuss during the interview? | Do you have any needs or desires for the birthing centre in terms of partnerships with clients? |

**Appendix 2. Interview Guide for Clients**

| Question | Questions for further development |
| --- | --- |
| Why did you choose to be followed in a birthing centre? | Is there a difference between a midwife follow-up and a hospital follow-up? |
| How would you characterize your relationship with your midwife or midwives? | - What do you consider to be most important in this relationship?  - Are you satisfied with your relationship with the midwives?  - How important do you consider the roles played by confidence, autonomy, shared decision making,  information sharing, adaptation of your follow-up to the context of your life, empathy and recognition of your expertise? |
| How would you describe your engagement/involvement in your pregnancy (delivery and postpartum)? | - Did you change any behaviors when you became pregnant? If yes, which ones and why?  - Did you go to all the proposed appointments? If not, why?  - Did you have to make any decisions related to your pregnancy, your delivery or your postpartum? If yes, what were they? |
| What do you see as the strengths and areas for improvement in the follow-up of pregnant and postpartum women in this birthing centre? | - Did you have to deal with any particular problem?  - What did you like most about your experience?  - Would you recommend this birthing centre to your best friend? If so, why? |
| How were you able to share your experience with the birthing centre? | - How is information on your experience collected?  - Do you know to whom you can turn if you have medical or non-medical problems?  - Do you feel that the concerns of women and their families are considered and included in the birthing centre? |
| What improvements do you think could be made for women and their families for a better experience at this birthing centre? | - What promotes and limits your level of involvement? |
| Are you involved in the birthing centre? | - Would you like to be more involved in the birthing centre?  - Do you feel that women and families can take ownership of the birthing centre with the midwives?  - How long have you been involved on the parents' committee?  - What led you to get involved on the parents' committee? What motivated you?  - What values of the parents’ committee are most important?  - What activities or actions do you participate in?  - Do you play a particular role on the parents' committee?  - Has your level of involvement in the birthing centre changed over time or through specific events?  - Can you identify any limitations or disincentives to the committee's actions?  - Can you identify any factors that support the committee's actions? |
| - Are you a member of the parents' committee? | If yes:  - How long have you been involved on the parents' committee?  - What led you to get involved on the parents' committee? What motivated you?  - What values of the parents' committee are most important?  - What activities or actions do you participate in?  - Do you play a particular role on the parents' committee?  - Has your level of involvement in the birthing centre changed over time or through specific events?  - Can you identify any limitations or disincentives to the committee's actions?  - Can you identify any factors that support the committee's actions? |
| Is there anything you would like to share that we did not discuss the interview? | - Do you have any needs and desires for the birthing centre? |

**Appendix 3. Interview Guide for the Manager**

| Question | Questions for further development |
| --- | --- |
| What is the relationship between the clients and the MWs/BAs at the birthing centre? | - What do you consider to be the most important aspect of this relationship?  - What do you make available to midwives to foster this relationship between the clients and their partners and the MWs/BAs? |
| Are the families involved in their care? Do you consider this important? | - What do you make available to families to enable them to get involved in their care?  - Do you encourage family autonomy? If yes, how?  - Do you encourage the sharing of information? If yes, how?  - Do you encourage shared decision-making? If yes, how?  - Do you encourage recognition of your clients' expertise? If yes, how?  - Do you encourage adaptation to clients’ living situation, desires and needs? If yes, how?  - What helps or hinders the level of client involvement at the birthing centre? |
| Can you explain how the parents' committee works? | - Why was the parents' committee set up?  - Who set up the parents' committee and how did it come about? How long has it been in place?  - How many parents are involved?  - What are the tasks of the parents' committee? |
| What resources are available to the parents' committee? | - Are there places or times when the parents' committee can express its point of view, give its opinion or take part in decisions made by the birthing centre?  - What is the relationship between the birthing centre and the parents' committee? |
| How are decisions made at the birthing centre? | Can families take part in decision-making? |
| Are there other ways for families to get involved in the birthing centre? | - How can families give their opinions on the birthing centre (its organization, etc.)?  - Who can they turn to for non-medical problems relating to the organization of care and services? |
| How do you define the care and service partnership with clients and their partners? | - Do you believe there is a care and service partnership between the clients, their partners and the professionals at the birthing centre?  - Do you want to encourage it?  - In your opinion, what are the areas for improvement and the obstacles to a partnership between families and midwives in a birthing center? |
| Is there anything you would like to share that we did not discuss during the interview? | Do you have any plans for involving families in birthing centres? |
